# Prenatal diagnosis and genetic counseling in a fetus associated with risk of Angelman syndrome with a small supernumerary marker chromosome derived from chromosome 22

**DOI:** 10.1186/s13039-016-0248-6

**Published:** 2016-05-03

**Authors:** Yu-an Hu, Yingxia Cui, Xiaobo Fan, Qiuyue WU, Weiwei Li, Weiping Wang

**Affiliations:** Institute of Laboratory Medicine, Jinling Hospital, Nanjing University School of Medicine, 305 East Zhongshan Road, Nanjing, Jiangsu 210002 China

**Keywords:** Angelman syndrome, Small supernumerary marker chromosomes (sSMC), Genetic counseling, Prenatal diagnosis

## Abstract

**Background:**

Angelman syndrome (AS) is a neurodevelopmental disorder. AS patients concomitant with sSMC are rather rare events. It will provide more useful and proper information for genetic counseling to identify the sSMC origin.

**Case presentation:**

A 27-year-old woman was referred for genetic counseling and prenatal diagnosis at 26 weeks of gestation due to her elder daughter, diagnosed as Angelman syndrome (AS) with an interstitial deletion in one of the chromosomes 15, carrying a small supernumerary marker chromosome (sSMC). The G-banding results of the woman and her current fetus both were 47,XX,+mar. In this paper, fluorescence in situ hybridization (FISH) results showed that there was no deletion of chromosome 15 in the woman and fetus. We demonstrated that the proband’s sSMC was maternally inherited and was an inv dup(22)(q11.1) , and that the deletion in 15q11.2-q13.1 was de novo.

**Conclusions:**

Taking into account above results and normal phenotypes of the proband’s mother, in this case we suggest that the sSMC don’t increase the recurrence risk of AS. After prenatal diagnosis, the woman chose to continue the pregnancy, and finally gave birth to a normal female infant.

## Background

Angelman syndrome (AS) is a neurodevelopmental disorder. Main clinical characteristics of AS include severe developmental delay, speech impairment, movement or balance disorder and apparent happy demeanor. Genetic mechanisms of AS involved the commonest, (micro) deletions in maternal chromosome 15q11-13, paternal uniparental disomy 15 (UPD), imprinting defects and/or mutation in the disease-causing gene *UBE3A* (ubiquitin protein ligase E3A) [OMIM:105830] [1].

Small supernumerary marker chromosomes (sSMC) are defined as structurally abnormal chromosomes that cannot be identified or characterized unambiguously by conventional banding cytogenetics alone. The size of sSMC is generally equal to or smaller than a chromosome 20 of the same metaphase spread [2]. The incidence has been estimated to be 0.075 % in unselected prenatal cases and 0.044 % in newborn infants, but evelated to 0.288 % in mentally retarded patients [3]. Furthermore, 70 % of sSMC are derived from acrocentric chromosomes [2].

As far as we know, there were rare nine AS cases reported in connection with sSMC. All AS cases with sSMC reported were related to chromosome 15. It is generally considered that the sSMC derived from chromosome 15 and contained the Prader–Willi/Angelman syndrome critical region (PWACR) would cause clinical phenotypes. We reported a previous case of AS with sSMC derived not from chromosome 15 [4]. At that time, we excluded the possibility of paternally- inherited origin of sSMC, but couldn’t deduce whether the origin of sSMC was de novo or maternally inherited. In this paper, the proband’s mother gave her consent to genetic testing and counselled for her fetus. In order to evaluate the recurrence risk of AS correctly, we made clear that the proband’s sSMC was maternally inherited and was an inv dup(22)(q11.1), and that the deletion in 15q11.2-q13.1 was de novo. Prenatal diagnosis and genetic counseling was performed in view of the findings.

## Case presentation

A 27-year-old woman was referred for genetic counseling and prenatal diagnosis at 26 weeks of gestation because her first daughter was diagnosed as Angelman syndrome (AS) due to a 5.058 Mb deletion in chromosome band 15q11.2-q13.1 and with a small supernumerary marker chromosome (sSMC). The proband was born by the woman and her former husband. Neither with her former husband nor with her current husband, they were in a non-consanguineous marriage. There was no family history of miscarriage or congenital malformations from either the two-term husbands or the woman. No abnormal symptoms were observed, and physical examination revealed that the couple was phenotypically normal.

The proband was diagnosed as AS at 3-year-old. G-banding revealed a karyotype 47,XX,+mar in all of the peripheral blood lymphocytes. Her paternal karyotype was normal and the information of her mother was unknown formerly. Silver staining for the nucleolus organizer regions (NOR staining) revealed two satellites in both ends of the sSMC. Fluorescence in situ hybridization (FISH) using the 15 dual color DNA probes (Vysis, USA), which hybridize to *D15S10,UBE3A* and centromere of chromosome 15 confirmed the deletion of chromosome 15q11-13 and the sSMC was unrelated with chromosome 15.

## Material and methods

### Cytogenetic analysis

Peripheral blood from the woman and cord blood from the fetus were drawn for cytogenetic analysis including G-banding and fluorescence in situ hybridization (FISH). FISH using the 15 dual color DNA probes (Vysis, USA) was performed according to standard procure. Centromeric FISH probes for chromosome 14 and 22, followed by sub-centromeric FISH probes for chromosome 14 or 22 were adopted to identify the the origin of sSMC in the proband. Centromere FISH and sub-centromere FISH were kindly performed by the laboratory of Professor Thomas Liehr (Jena University Hospital, Friedrich Schiller University, Institute of Human Genetics).

## Results

**Fig. 1:**
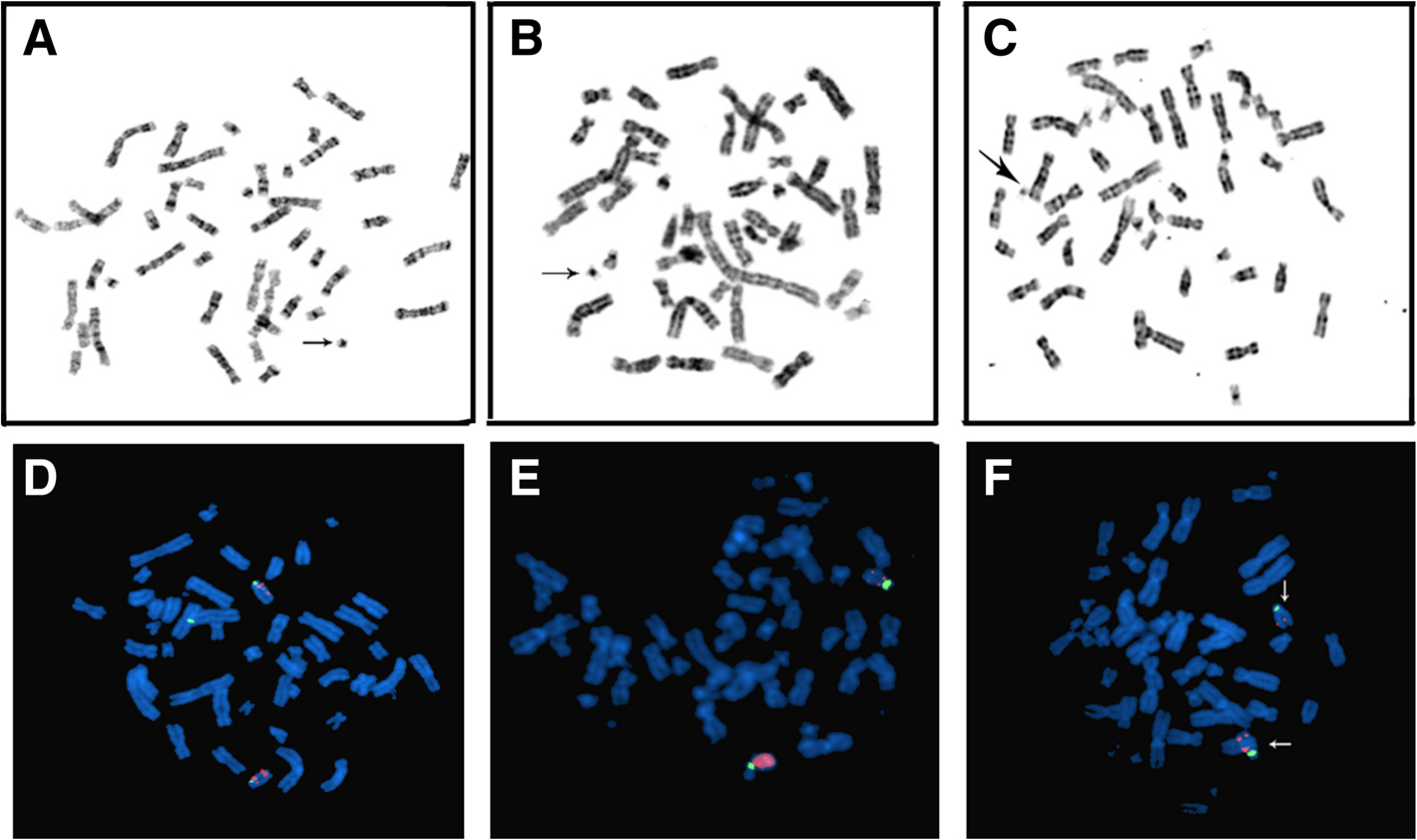
Results of karyotypes and FISH using the 15 dual color DNA probes. **a**–**c**: The results of karyotypes from the mother (**a**), the fetus (**b**) and the proband (**c**) showed the sSMC in the proband and the fetus were maternally inherited. The *black arrow* indicates the sSMC. **d**–**f**: The results of FISH using the 15 dual color DNA probes showed that there was no deletion in chromosome 15q11-13 observed both in the mother (**d**) and in the fetus (**e**) and that there was no additional centromeric signal of chromosome 15 in the three cases. The *white arrow*(↓) shows the loss of chromosome 15q11-13 and the *white arrow to left* (←) indicates normal chromosome 15 in the proband (**f**). The *orange* signals indicate chromosome 15q11-13 and 15q22-24(used as control probe), the green signal indicates the centromere of chromosome 15. **a**, **d**: the mother of proband; **b**, **e**: the fetus; **c**, **f**: the proband

G-banding showed both of karyotypes in woman and her fetus were 47,XX,+mar in 100 % of the analyzed cells.. This revealed the small supernumerary marker chromosome (sSMC) in the proband was maternally inherited. Fluorescence in situ hybridization confirmed that there was no deletion of chromosome 15q11-13 in the woman and her fetus, therefore Angelman syndrome was not considered. FISH analysis with centromeric probe of chromosome 15 also excluded the possibility of the origin from the chromosome 15 (Fig. 1).

**Fig. 2:**
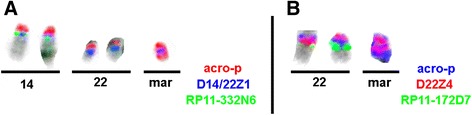
The origin of marker chromosome characterized by centromeric FISH and subcentromeric FISH. **a**: The sSMC wasn’t derived from chromosome 14. The centromeric probe specific for chromosome 14 / 22(D14/22Z1 *blue*) and a probe specific for all acrocentric p-arms (acro-p *red*) were present on the marker chromosome. But the centromere-near probe in 14q11.2(RP11-332 N6 *green*) was not detected on the marker chromosome. **b**: The sSMC originated from chromosome 22 and was an inv dup(22)(q11.1). The centromeric probe specific for chromosome 22(D22Z4 red) and a probe specific for all acrocentric p-arms (acro-p *blue*) were present on the marker chromosome. A centromere-near probe in 22q11.21(RP11-172D7 *green*) was not detected.14:chromosome 14; 22: chromosome 22; mar: sSMC

Centromeric FISH and sub-centromeric FISH on the proband’s metaphases spreads demonstrated that the sSMC originated from chromosome 22 and was a variant of inv dup(22)(q11.1) (Fig. 2), thus the final karyotype of the proband was written as 47,XX,del(15)(q11q13),+inv dup(22)(q11.1).

## Discussion

Angelman syndrome (AS) has a prevalence of 1/10,000 ~ 1/20,000 individuals [1], it could be clinically diagnosed by a serial of characteristic features including developmental delay, intellectual disability, speech impairment, seizures, movement or balance disorder, apparent happy demeanor, and so on. Genetic analysis, such as karyotype, fluorescence in situ hybridization (FISH), methylation analysis of the chromosome 15q11-13 region and chromosomal microarrays analysis might be helpful for the diagnosis of AS and provide information as much as possible for genetic counseling. In our previous report, Affymetrix cytogenetics whole genome 2.7 M arrays were applied to demonstrate a 5.058 Mb deletion in chromosome band 15q11.2-q13.1 between positions chr15: 21,170,573-26,229,285 bp, in which 10 genes (*SNORD116-14, SNORD115-30, UBE3A, SNORD109B, SNORD115-23, SNORD116-9, SNORD116-16, SNORD115-10, GABRG3, SNORD116-2)* were contained. The microarrays analysis further characterized the region confirmed by FISH.

What’s special about the AS proband we reported was accompanied by a small supernumerary marker chromosome (sSMC). AS patients concomitant with sSMC are rather rare events. Just nine AS cases with the sSMC were reported by now. As summarized in sSMC-homepage (http://ssmc-tl.com/sSMC.html), the corresponding sSMC were derived from chromosome 15 in all nine cases. In five of them, the AS was due to a paternal UPD 15, in three due to a deletion in the AS-critical region [5–8] and in the remainder case the molecular result for AS was not elucidated. Chromosome 15 is the most common origin of sSMC in human [2, 3]. Summarizing data indicated that the majority of sSMCs (65 %) originated from chromosome 15, while sSMCs derived from other acrocentric chromosomes 13, 14, 21, and 22 constitute only 7 % [9]. When AS accompanied by sSMC, particularly contained the Prader–Willi/Angelman syndrome critical region in chromosome 15, the correlation between the genotype and the clinical phenotype became more complex [10]. The abnormal phenotype was associated with the origin and genetic nature of sSMC. It is important to identify the chromosome origin if the sSMC is visible in the karyotype of AS.

In our case, karyotypes showed the sSMC was maternally inherited. Furthermore, FISH with centromeric probe of chromosome 15 revealed that the sSMC was unrelated to chromosome 15. Finally, Centromeric FISH and sub-centromeric FISH were carried out to confirm that the sSMC derived from chromosome 22 and was an inv dup(22)(q11.1) (Fig. 2). Additionally, data from Affymetrix cytogenetics whole genome 2.7 M arrays did not indicate the origin information. We speculated that the genetic material nature of the sSMC was heterochromatin and there was no annotated gene present corresponding to the arrays. Thus, the proband we reported previously, to our knowledge, is the first AS case with the maternally inherited sSMC derived from chromosome 22.

The risk to the sibs of an individual with AS who was identified a *de novo* large deletion in the chromosome 15q11-13 is reported < 1 % [11]. However, it is estimated that there were about 2.7x10^6^ living sSMC carriers in the world, almost 70 % of those are clinically normal [12]. Furthermore, in 70 % of the carriers the sSMC is de novo, in 20 % inherited from the mother, and in 10 % inherited from the father. Familial sSMCs in general have little or no effect on the phenotype if the parent’s development is normal [13].

Taking into account the normal phenotype of the proband’s mother, we thought that AS in the proband could be explained by the microdeletion on one of the chromosome 15q11-13, and not by the presence of the sSMC.

After prenatal diagnosis and genetic counseling, the woman chose to continue the pregnancy, and finally gave birth to a normal female infant.

## Conclusion

In summary, the recurrence risk of AS depends on the genetic mechanism of AS in the proband. When AS accompanied by sSMC, it will provide more useful and proper information for genetic counseling to identify the sSMC origin.

### Consent

Written informed consent was obtained from the patient for publication of this case report and any accompanying images. A copy of the written consent is available for review by the Editor-in-Chief of this journal.
